# Identification of heart failure with preserved ejection fraction helps risk stratification for hypertrophic cardiomyopathy

**DOI:** 10.1186/s12916-021-02219-7

**Published:** 2022-01-26

**Authors:** Jie Liu, Dong Wang, Jieyun Ruan, Guixin Wu, Lianjun Xu, Wen Jiang, Jizheng Wang, Xiaolu Sun, Lianming Kang, Lei Song

**Affiliations:** 1 State Key Laboratory of Cardiovascular Disease, Fuwai Hospital, National Center for Cardiovascular Diseases, Chinese Academy of Medical Sciences and Peking Union Medical College, 167, Beilishi Road, Xicheng District, 100037 Beijing, People’s Republic of China; 2 Cardiomyopathy Ward, Fuwai Hospital, National Center for Cardiovascular Diseases, Chinese Academy of Medical Sciences and Peking Union Medical College, Beijing, 100037 People’s Republic of China; 3 National Clinical Research Center for Cardiovascular Diseases, Fuwai Hospital, National Center for Cardiovascular Diseases, Chinese Academy of Medical Sciences and Peking Union Medical College, Beijing, 100037 People’s Republic of China

**Keywords:** Hypertrophic cardiomyopathy, Heart failure, Preserved ejection fraction, Outcomes

## Abstract

**Background:**

Heart failure with preserved ejection fraction (HFpEF) is the dominant form of heart failure (HF). We here aimed to investigate the characteristics and prognosis of HFpEF in patients with hypertrophic cardiomyopathy (HCM).

**Methods:**

This was a prospective cohort study and patients with HCM with available NT-proBNP results were enrolled. Patients were categorized into HFpEF [defined as LVEF ≥50%, with symptoms or signs of HF, and N-terminal pro-brain natriuretic peptide ≥800 pg/mL according to American Heart Association (AHA) criteria] and without heart failure (non-HF). The outcomes of interest were all-cause death, cardiovascular death, and sudden cardiac death (SCD).

**Results:**

Of 1178 included patients with HCM, 513 (43.5%) were identified as having HFpEF according to AHA criteria. Compared with non-HF patients, patients with HFpEF had significantly larger maximal wall thickness (*P* < 0.001), higher maximal left ventricular outflow tract gradient (*P* < 0.001), higher proportion of atrial fibrillation (*P* < 0.001), higher incidence of all-cause death (log-rank test, *P* = 0.002), and cardiovascular death (log-rank test, *P* = 0.005). Multivariable Cox analysis showed that patients with HFpEF had a nearly two-fold higher risk of all-cause death (adjusted HR = 1.80, 95% CI 1.11–2.90; *P* = 0.017) and cardiovascular death (adjusted HR =1.82, 95% CI 1.05–3.18; *P* = 0.033) than non-HF patients.

**Conclusions:**

Patients with HCM have a high prevalence of HFpEF and those with HFpEF present greater disease severity and higher mortality than non-HF patients, and thus may require an appropriate and more aggressive treatment for HF management. Identification of patients with HFpEF using AHA criteria can provide guidance on patient risk stratification for patients with HCM.

**Supplementary Information:**

The online version contains supplementary material available at 10.1186/s12916-021-02219-7.

## Background

Hypertrophic cardiomyopathy (HCM) is one of the commonest myocardial diseases, its estimated prevalence being 1/500–1/200 [1]. Heart failure (HF) is a major cause of mortality in patients with HCM. Recognition of HF is important because it influences patient management. Previous studies of HCM focused on patients with systolic dysfunction [left ventricular ejection fraction (LVEF) < 50%] because those patients reportedly have a high risk of HF-related death and sudden cardiac death (SCD) [2]. However, left ventricular systolic dysfunction usually appears in end-stage of disease, following with ventricular dilatation and a thin ventricular wall. Previous studies have shown that patients with LVEF < 50% account for only 2–8% of all patients with HCM and that these patients usually require advanced therapies, including implantable cardioverter-defibrillators and heart transplants [2–4]. Thus, systolic function is preserved in most patients with HCM.

Clinical practice guidelines of HF proposed the concept of HF with preserved ejection fraction (HFpEF) [5, 6]. Previous studies have shown that HFpEF is the dominant form of HF in patients with cardiovascular disease and is associated with poor quality of life and premature mortality [7]. To the best of our knowledge, no published study has yet described the clinical characteristics of HFpEF in patients with HCM. Whether recognizing HFpEF will assist risk stratification or have treatment implications for patients with HCM remains to be fully examined. Moreover, current guidelines recommend identifying HFpEF on the basis of the presence of symptoms and/or signs of HF, LVEF≥50%, high natriuretic peptides concentrations, and the presence of relevant structural heart disease and/or diastolic dysfunction. However, the cutoff value for high natriuretic peptides concentrations remains controversial. The 2016 European Society of Cardiology (ESC) guideline recommends cutoff values of B-type natriuretic peptide (BNP) > 35 pg/mL and/or N-terminal pro-brain natriuretic peptide (NT-proBNP) > 125 pg/mL, whereas the 2017 American Heart Association (AHA) scientific statement suggests using BNP ≥100 pg/mL or NT-proBNP ≥800 pg/mL as the cutoff values for a diagnosis of HFpEF [6, 8]. Which cutoff value more reliably identifies patients with HFpEF is yet to be investigated in patients with HCM. Thus, we conducted this study of a large cohort of patients with HCM with the aims of determining the characteristics and prognosis of HFpEF in patients with HCM and of evaluating which cutoff value of NT-proBNP is better for recognizing HFpEF in patients with HCM.

## Methods

### Study cohort

The cohort of this observational cohort study comprised 1238 unrelated, prospectively enrolled patients with HCM with available NT-proBNP results at enrolment. Patients with HCM were consecutively recruited, including both outpatients and inpatients that were admitted to hospital for various reasons. Thirty-six of these patients were excluded from the study because they were lost to follow-up after enrolment and 24 patients were excluded because of with LVEF < 50%, leaving 1178 patients with HCM for inclusion in the analysis. All patients were prospectively enrolled at Fuwai Hospital, Chinese Academy of Medical Sciences, between 1999 and 2019. HCM was identified by echocardiographic and/or cardiac magnetic resonance demonstration of left ventricular hypertrophy (maximal left ventricular wall thickness ≥15 in general or ≥13 mm in patients with a family history of HCM) in the absence of any other cardiac or systemic disease capable of producing such severe hypertrophy. Written informed consent was obtained from all study patients. The study was performed in accordance with the principles of the Declaration of Helsinki and was approved by the Ethics Committee of Fuwai Hospital.

All patients underwent a complete cardiac evaluation on enrollment. LVEF was calculated from two-dimensional echocardiography images using the modified Simpsons rule formula. NT-proBNP concentrations were measured as described in our previous study [9]. Specifically, venous blood samples were collected into serum separator tubes by direct venipuncture and sent to the laboratory for testing. Serum NT-proBNP concentrations were measured using a commercially available, fully automated, two-side electrochemiluminescence immunoassay (Cobas E170, Roche, Basel, Switzerland).

### Recognition of HFpEF

Diagnosis of HFpEF was based on a combination of symptoms, signs, LVEF and NT-proBNP concentrations. According to the 2017 AHA criteria [8], patients with LVEF ≥50%, NT-proBNP ≥800 pg/mL, and with symptoms or signs of HF were categorized as having HFpEF, and those who did not meet the above criteria were identified as having non-HF. In contrast, the 2016 ESC guideline suggested using NT-proBNP>125 pg/mL as the cutoff point for a diagnosis of HFpEF [6].

### Genotyping

Genetic testing using genomic DNA extracted from a blood sample was performed on 946 (80.3%) patients in the study cohort, 692 of whom underwent whole exome sequencing and 254 panel sequencing. Variants identified in eight core sarcomeric protein-encoding genes (*MYH7*, *MYBPC3*, *TNNT2*, *TNNI3*, *MYL2*, *MYL3*, *TPM1*, and *ACTC1*) were classified as pathogenic, likely pathogenic, of unknown significance, likely benign or benign using the criteria proposed by the American College of Medical Genetics and Genomics [10]. Patients with any pathogenic or likely pathogenic variant in these eight genes were grouped as mutation-positive, whereas those without any such variants were grouped as mutation-negative.

### Outcomes

All patients were followed up annually until July 2020 by a clinic visit or telephone interview. Patients lost to follow-up were censored at the last known contact date. The outcomes of interest were all-cause death, cardiovascular death, and sudden cardiac death (SCD). SCD events were a composite of SCD and equivalent events. SCD was defined as witnessed sudden and unexpected death with or without documented ventricular fibrillation within 1 h of new symptoms developing or nocturnal death with no history of worsening symptoms. Resuscitation from cardiac arrest and appropriate implantable cardioverter-defibrillator shock therapy for ventricular tachycardia or fibrillation were considered to be equivalent to SCD.

### Statistical analysis

Categorical variables were presented as numbers and percentages and continuous variables as means and standard deviations or medians with interquartile ranges. Differences in characteristics across different groups were compared with the *χ*^2^ test for categorical variables and Student’s *t* test or Mann-Whitney *U* test for continuous variables. Survival curves were constructed by the Kaplan-Meier method and compared by the log-rank test. Univariable and multivariable Cox proportional hazards models were used to calculate hazard ratios (HRs) and 95% confidence intervals (CIs). Multivariable Cox regression analysis was conducted by adjusting the following confounders using a backward method: age, sex, maximal left ventricular wall thickness, atrial fibrillation, maximal left ventricular outflow tract gradient, and NYHA class. Receiver operating characteristic (ROC) curve was constructed to visualize the risk prediction performances by plotting the sensitivity against 1-specificity. Youden’s index was the sum of sensitivity and specificity minus one, being a commonly used means of determining the optimal threshold. In consideration that septal reduction therapy could significantly affect patient clinical course and outcomes, we performed sensitivity analysis by excluding patients with septal reduction therapy. A two-tailed *P* value of ≤0.05 was considered to denote statistical significance. All statistical analyses were performed using SPSS version 24 (IBM, Armonk, NY) and GraphPad Prism 8.0.1.

## Results

### Clinical characteristics of the study cohort

Of the 1178 patients included, 513 (43.5%) patients were identified as having HFpEF using AHA criteria. The patients’ baseline characteristics according to HF phenotypes were summarized in Table 1. Compared with non-HF patients, patients with HFpEF were more often female (44.6% versus 28.0%; *P* < 0.001) and had significantly greater maximal wall thickness (24 mm versus 22 mm; *P* < 0.001), higher maximal left ventricular outflow tract gradient (64 mmHg versus 25 mmHg, *P* < 0.001), higher NT-proBNP concentrations (1807.0 pg/mL versus 536.8 pg/mL; *P* < 0.001) and higher proportion of atrial fibrillation (27.3% vs. 13.5%; *P* < 0.001). Use of the diagnostic criterion of HFpEF in the ESC guidelines yielded 761 (64.6%) patients with HFpEF and similar significant differences were observed between non-HF patients and patients with HFpEF (Additional file 1: Table. S1).

**Table 1:** Clinical characteristics of patients with HFpEF and non-HF patients in HCM

Parameters	All (*n*=1178)	HFpEF^#^ (*n*=513)	Non-HF^#^ (*n*=665)	*P* value^*^
Age at evaluation (years)	49±14	50±14	48±14	0.146
Female	415 (35.2)	229 (44.6)	186 (28.0)	< 0.001
MWT (mm)	23 (20–26)	24 (21–28)	22 (19–25)	< 0.001
LVEF (%)	68±8	69±7	68±6	0.027
LVedd (mm)	44±6	43±6	44±5	< 0.001
Unexplained syncope	149 (12.6)	75 (14.6)	74 (11.1)	0.070
Atrial fibrillation	230 (19.5)	140 (27.3)	90 (13.5)	< 0.001
Maximal LVOT gradient	44 (10–80)	64 (29–94)	25 (8–67)	< 0.001
NT-proBNP	988.0 (494.1–1934.4)	1807.0 (1225.0–2892.6)	536.8 (304.6–796.8)	< 0.001
NYHA class				
I	385 (32.7)	0 (0.0)	385 (57.9)	< 0.001
II	514 (43.6)	313 (61.0)	201 (30.2)	< 0.001
III/IV	279 (23.7)	200 (39.0)	79 (11.9)	< 0.001
Medicine treatment				
Beta-blockers	861 (73.1)	387 (75.4)	474 (71.3)	0.110
ACEI/ARBs	232 (19.7)	70 (13.6)	162 (24.4)	< 0.001
Calcium-channel blocker	230 (19.5)	87 (17.0)	143 (21.5)	0.051
Diuretic	269 (22.8)	160 (31.2)	109 (16.4)	< 0.001

In our cohort, the data on the maximal LVOT gradient were not available in 15 (1.3%) patients and the missing values were imputed using the median.

Values are presented as the mean±SD, median (interquartile range) or *n* (%)

*AHA* American Heart Association, *LVedd* left ventricular end-diastolic dimension, *LVEF* left ventricular ejection fraction, *LVOT* left ventricular outflow tract, *MWT* maximal wall thickness, *NT-proBNP* N-terminal pro-brain natriuretic peptide, *NYHA* New York Heart Association

^#^Heart failure phenotypes were identified using AHA criteria

*Comparison between patients with HFpEF and non-HF patients

### Cumulative incidence of adverse events according to HF phenotypes

**Fig. 1:**
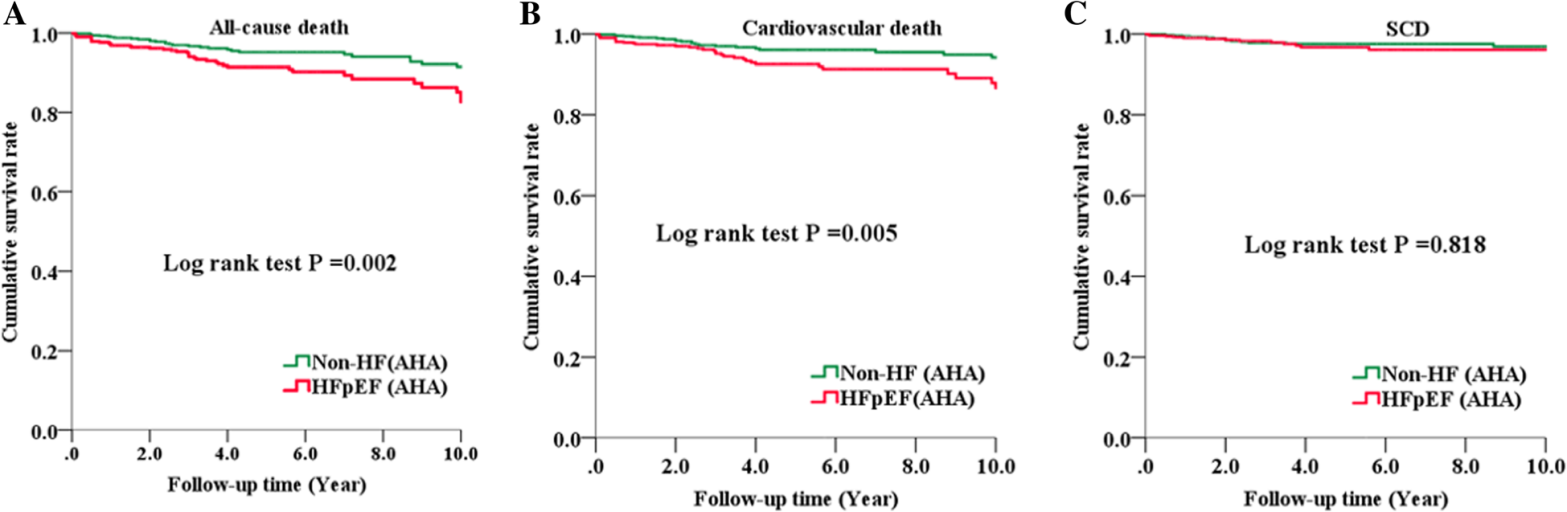
Kaplan-Meier curve for patients with HFpEF and non-HF patients using AHA criteria in HCM. Cumulative survival rate curves for all-cause death (**A**), cardiovascular death (**B**), and SCD (**C**). AHA, American Heart Association; HCM, hypertrophic cardiomyopathy; HFpEF, heart failure with preserved ejection fraction; non-HF, patients without heart failure; SCD, sudden cardiac death

Over a total follow-up of 5827 patient years, all-cause death occurred in 79 patients (1.4 per 100 patient years). Sixty of these events were cardiovascular deaths, including 29 SCD events. The incidence of all-cause death and cardiovascular death in patients with HFpEF were 1.92 and 1.51 per 100 patient-years, whereas only 0.96 and 0.70 per 100 patient-years in non-HF patients. Kaplan-Meier survival analysis demonstrated a significant difference in the cumulative event-free survival rate of all-cause death (*P* =0.002) and cardiovascular death (*P* =0.005) between patients with HFpEF and non-HF patients (Fig. 1). The incidence of SCD was 0.54 per 100 patient-years in the subgroup of patients with HFpEF, which is similar to that in non-HF patients (0.47 per 100 patient-years). In contrast, when we used ESC criteria for the diagnosis of HFpEF, no significant difference was observed in the incidence of all-cause death, cardiovascular death or SCD between the patients with HFpEF and non-HF patients (Additional file 2: Fig. S1).

### Independent association between HFpEF and poor prognosis

**Fig. 2:**
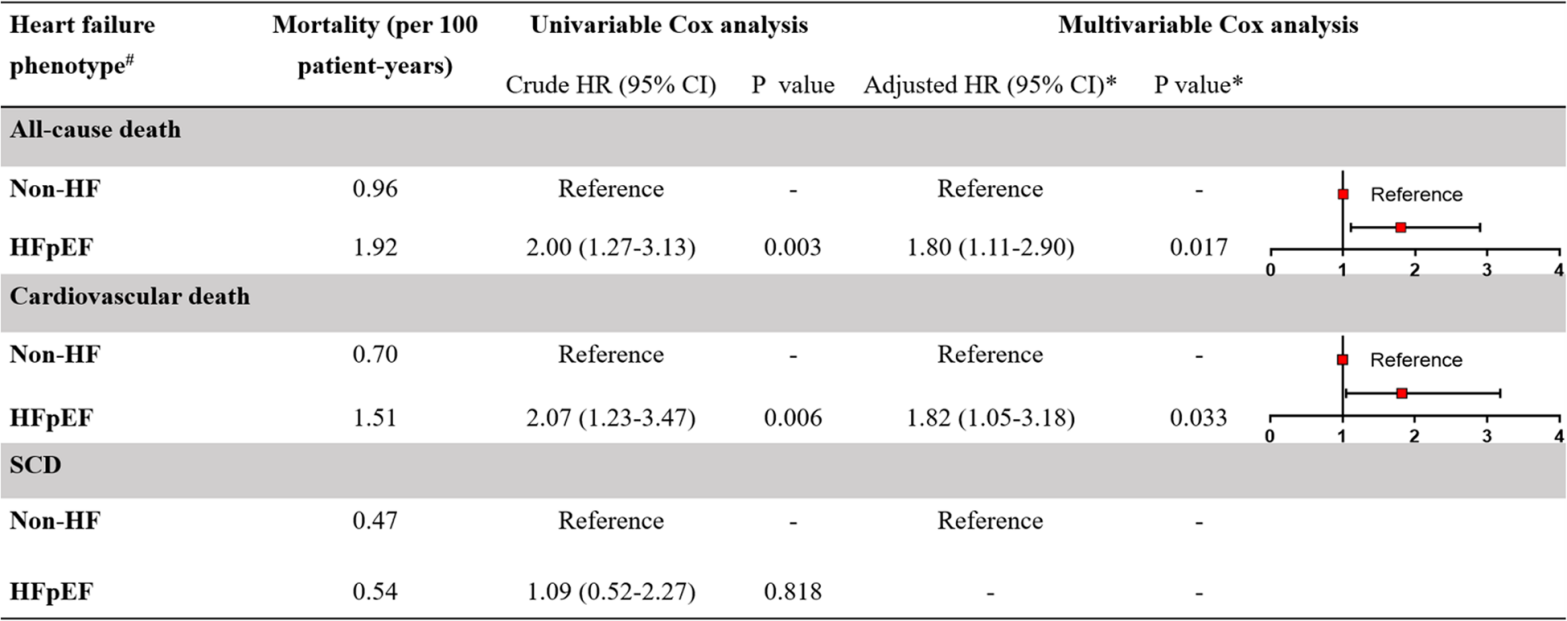
Hazard ratio of HFpEF versus non-HF in 1178 patients with HCM. ^#^Heart failure phenotypes were identified using AHA criteria. *Multivariable Cox regression analysis, models were adjusted for the following covariates using a backward method: age, sex, maximal left ventricular wall thickness, atrial fibrillation, maximal left ventricular outflow tract gradient, and NYHA class. AHA, American Heart Association; CI, confidence interval; HFpEF, heart failure with preserved ejection fraction; non-HF, patients without heart failure; HR, hazard ratio; SCD, sudden cardiac death

**Fig. 3:**
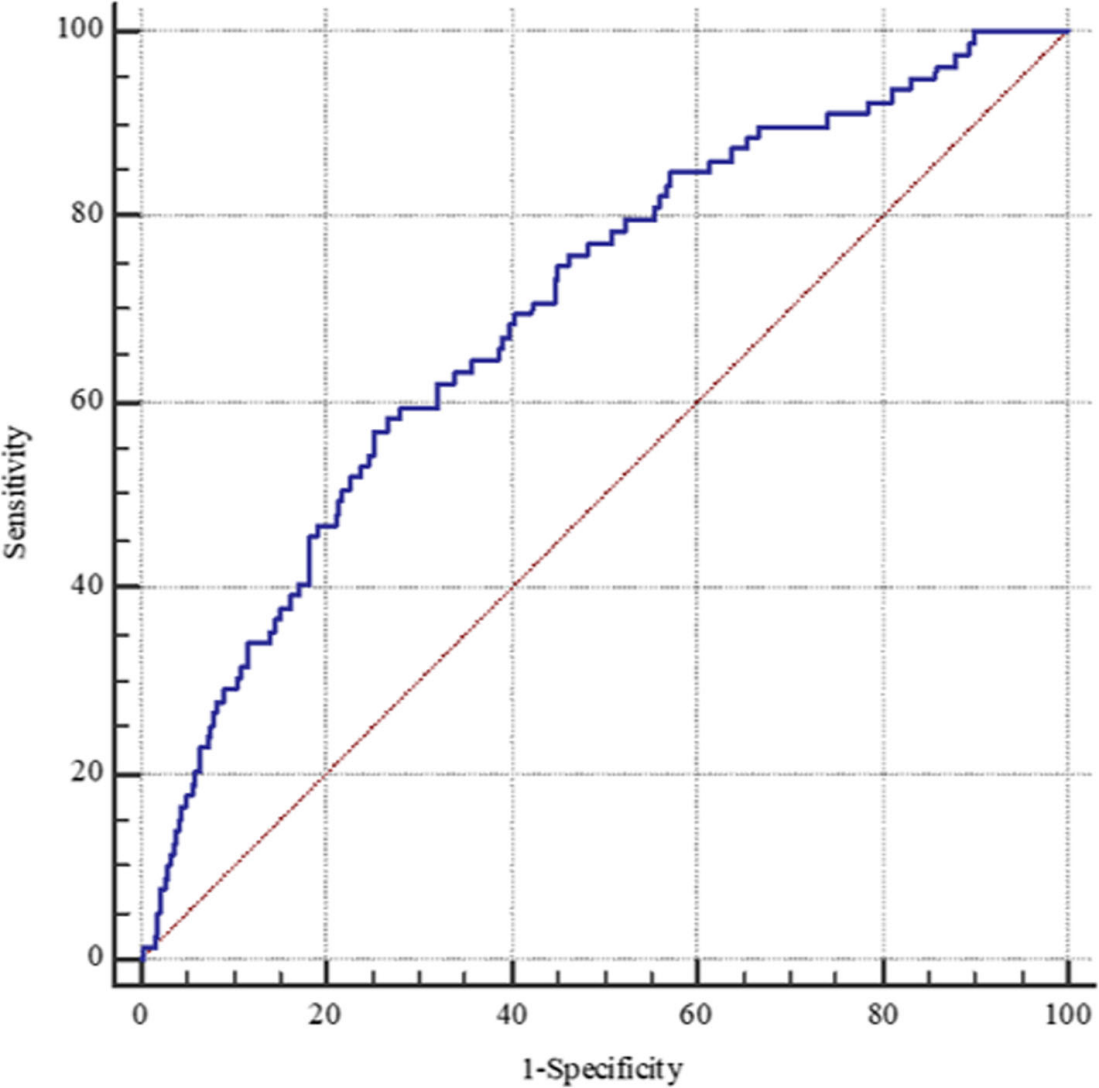
ROC curve of NT-proBNP for all-cause death risk prediction in 1178 patients with HCM. C-statistics = 0.70 (95% CI 0.67–0.73, *P* < 0.001); the optimal cut-off value was NT-proBNP > 1780 pg/ml identified by the highest Youden’s index (sensitivity =57.0%, specificity =74.8%). HCM, hypertrophic cardiomyopathy; NT-proBNP, N-terminal pro-brain natriuretic peptide; ROC, receiver operating characteristic

Univariable Cox regression analysis showed that, compared with non-HF, HFpEF was associated with a significantly higher risk of all-cause death (HR =2.00, 95% CI 1.27-3.13; *P* =0.003) and cardiovascular death (HR =2.07, 95% CI 1.23–3.47; *P* =0.006). Multivariable Cox analysis was conducted and the analysis showed that, compared with non-HF patients, those with HFpEF had a nearly twofold higher risk of all-cause death (adjusted HR =1.80, 95% CI 1.11–2.90; *P* =0.017) and cardiovascular death (adjusted HR =1.82, 95% CI 1.05–3.18; *P* =0.033). The results demonstrated an independent association between HFpEF and poor prognosis in the patients with HCM (Fig. 2). We further constructed a ROC curve to evaluate the predicting performance of various NT-proBNP cut-off value for all-cause death in patients with HCM (Fig. 3). The results showed that NT-proBNP > 1780 pg/ml had the highest Youden’s index. When we used the ESC criteria to guide the diagnosis of HFpEF, no significant association was found between HFpEF and prognosis in patients with HCM (Additional file 2: Fig. S2 ).

### Sensitivity analysis

Septal reduction therapy was performed after enrollment and clinical evaluation in 565 (48.0%) of the patients in our study cohort. We therefore conducted sensitivity analysis to exclude the potential effect of septal reduction therapy on patient outcomes and to assess the stability of our results. The study cohort analyzed included 613 patients with HCM who did not undergo septal reduction therapy; their results were similar to those of whole cohort. Multivariable Cox analysis showed that patients with HFpEF were at significantly higher risk of all-cause death (adjusted HR = 2.10, 95% CI 1.20–3.66; *P* =0.009) and cardiovascular death (adjusted HR = 2.21, 95% CI 1.14–4.27; *P* =0.019) than were non-HF patients (Additional file 2: Fig. S3). When we used the ESC criteria, no significant association was observed between HFpEF and prognosis (Additional file 2: Fig. S3).

### Genetic characteristics of HFpEF in HCM

Among the 946 patients in this cohort who underwent genetic testing, 307 (32.5%) were found to have a variant classified as “pathogenic” or “likely pathogenic” in the sarcomere gene, variants in *MYBPC3* and *MYH7* being predominant (Table 2). Six (0.6%) patients carried a combination of two pathogenic or likely pathogenic variants. Table 2 summarizes the gene mutation data for patients with HFpEF and non-HF patients. We found that the proportion of mutation-positive patients was significantly higher in the HFpEF group than in the non-HF group (36.6% vs. 30.3%, *P* =0.041).

**Table 2:** Genetic characteristics of patients with HFpEF in HCM

Genetics	All (*n*=946)	HFpEF^#^ (*n*=415)	Non-HF^#^ (*n*=531)	*P* value*
Mutation-positive	313 (33.1)	152 (36.6)	161 (30.3)	0.041
Multiple variants	6 (0.6)	4 (1.0)	2 (0.4)	0.413
*MYH7*	151 (16.0)	74 (17.8)	77 (14.5)	0.165
*MYBPC3*	127 (13.4)	56 (13.5)	71 (13.4)	0.956
*ACTC1*	0 (0.0)	0 (0.0)	0 (0.0)	-
*MYL2*	6 (0.6)	4 (1.0)	2 (0.4)	0.413
*MYL3*	4 (0.4)	3 (0.7)	1 (0.2)	0.325
*TNNI3*	16 (1.7)	8 (1.9)	8 (1.5)	0.618
*TNNT2*	13 (1.4)	9 (2.2)	4 (0.8)	0.064
*TPM1*	2 (0.2)	1 (0.2)	1 (0.2)	1.000

*AHA* American Heart Association, *HCM* hypertrophic cardiomyopathy, *HFpEF* heart failure with preserved ejection fraction, *non-HF* patients without heart failure

^#^Heart failure phenotypes were identified using AHA criteria

*Comparison between patients with HFpEF and non-HF patients

## Discussion

In the present study, we comprehensively investigated the prevalence, clinical and genetic characteristics, and clinical outcomes of patients with HCM with HFpEF. We found that HFpEF showed a high prevalence in HCM. These patients presented with more severe cardiac abnormalities and had a significantly worse prognosis than did non-HF patients. Moreover, the cutoff value for NT-proBNP of > 800 pg/mL was better for determining HFpEF than was the cutoff value of NT-proBNP ≥125 pg/mL in that it improved risk stratification in our patients with HCM.

Among patients with HCM, establishing a diagnosis of HF and determining the HF phenotype is not only important from the perspective of prognosis but may also have important treatment implications. Practice guidelines of HF proposed the concept of HFpEF, but to the best of our knowledge, no published studies have previously investigated the characteristics of HFpEF in patients with HCM. We therefore conducted this study with the aims of assessing the prevalence, clinical and genetic profiles, and long-term prognosis of patients with HFpEF in HCM.

The current criteria for diagnosis of HFpEF are inconsistent between different practice guidelines for HF, the main discrepancy being the cutoff values for high natriuretic peptides concentrations. The 2016 ESC guidelines recommend NT-proBNP > 125 pg/mL as the cutoff value, whereas the 2017 AHA scientific statement suggests using NT-proBNP ≥800 pg/mL. Further research is needed to determine whether the ESC criteria or the AHA criteria are more optimal for classifying patients with HCM. We found that the clinical outcomes of patients with HFpEF and non-HF patients did not differ significantly when we used the ESC criteria. In contrast, we found significant differences in risks of all-cause death and cardiovascular deaths between patients with HFpEF and non-HF patients when we used the AHA criteria. These findings imply that using the AHA criteria to identify patients with HFpEF is more helpful for risk stratification of patients with HCM.

We also found a high prevalence of HFpEF in patients with HCM. When we used the diagnostic criteria in the 2017 AHA scientific statement, we found that over 40% of the patients in our cohort fell into the HFpEF category. Additionally, the clinical characteristics of patients with HFpEF and non-HF patients differed significantly. Compared with non-HF patients, those with HFpEF had more severe cardiac abnormalities, as evidenced by a significantly larger maximal wall thickness, higher proportion of atrial fibrillation, higher maximal left ventricular outflow tract gradient, etc. These findings imply that a diagnosis of HFpEF may be a reliable indicator of disease progression in patients with HCM.

Most importantly, we found that patients with HFpEF were at a significantly higher risk of all-cause death and cardiovascular death than were non-HF patients. This difference remained significant after adjusting for potential confounders. We further conducted a sensitivity analysis to exclude the potential effects of septal reduction therapy on our finding. Similar results were observed when only including patients without septal reduction therapy, indicating that our results are robust. These findings indicate that patients with HCM with HFpEF have an elevated risk of premature mortality. Meanwhile, future studies could use HFpEF as a surrogate for hard endpoints in patients with HCM, thus reducing the length of the study period.

To date, several large-scale clinical trials have assessed the efficacy of medical therapies for HFpEF, jet no drug has been proven to reduce mortality [11]. Current HF guidelines recommend diuretics as the first-line treatment for patients with HFpEF to relieve symptoms due to volume overload, because previous studies have provided evidence to support the efficacy of diuretics in reducing the risk of hospitalization for HF [12]. There is also evidence supporting that aldosterone receptor antagonists and angiotensin-receptor blockers can decrease HF hospitalizations for patients with HFpEF [13–15]. Patients with HCM and HFpEF may also benefit from appropriate HF management by using drugs such as diuretics and spironolactone, although no direct evidence is available to support this at present time. Future large-scale randomized controlled trials for HFpEF treatment are warranted in patients with HCM.

Our findings show that the patients with HFpEF are not at increased risk of SCD. Previous studies have shown that LVEF < 50% is a strong indicator for a high risk of SCD in patients with HCM [2, 4, 16]. In contrast, the incidence of SCD events in patients with HFpEF was low and similar to that in non-HF patients. Instead of SCD prevention, the main goals of treatment for patients with HCM with HFpEF should be prevention/slowing of disease progression.

## Study limitation

This large study was conducted in a single national referral center. Although patients were from across the country, there may have been a selection bias. Meanwhile, possible bias due to long-time span of patient enrollment cannot be completely ruled out because there was some change in diagnosis and treatment of HCM over past years. Second, high creatine concentrations, kidney failure, and hyponatremia are reportedly associated with high NT-proBNP concentration. Only 13 (1.1%) of our participants had high creatine concentrations (> 133 μmol/L), 84 (7.0%) participants low eGFR concentrations [< 60 mL/(min*1.7m^2^)] and 18 (1.5%) hyponatremia (serum sodium < 135 mmol/L). The current cut-off value for NT-proBNP may be of limited value in diagnosing HFpEF in such patients with HCM. Thus, the optimal cut-off value still needs further investigation.

## Conclusion

In patients with HCM, HFpEF is common, can reflect the state of disease progression, and is independently associated with increased risk of all-cause death and cardiovascular death, but not with an increased risk of SCD. Moreover, the cut-off values of NT-proBNP for recognizing HFpEF in AHA criteria better stratify risk in a patient with HCM than those in the ESC criteria.

## Supplementary Information

**Additional file 1: Table S1**. Clinical characteristics of patients with HFpEF and non-HF patients using ESC criteria in HCM.

**Additional file 2: Figure S1-S3**. **FigS1**- Kaplan-Meier curve for patients with HFpEF and non-HF patients using ESC criteria in HCM. **FigS2**- Hazard ratio of HFpEF versus non-HF using ESC criteria in 1178 patients with HCM. **Fig S3**-Hazard ratio of HFpEF versus non-HF in 613 HCM patients without septal reduction therapy.

## Data Availability

All authors had full access to all data in the study and take responsibility for the integrity of data and the accuracy of data analysis.

## Abbreviations

AHA: American heart association
BNP: B-type natriuretic peptide
CI: Confidence intervals
ESC: European society of cardiology
HCM: Hypertrophic cardiomyopathy
HF: Heart failure
HFpEF: Heart failure with preserved ejection fraction
HR: Hazard ratios
LVEF: Left ventricular ejection fraction
NT-proBNP: N-terminal pro-brain natriuretic peptide
SCD: Sudden cardiac death
