# Supplementary material for: Identification of heart failure with preserved ejection fraction helps risk stratification for hypertrophic cardiomyopathy
Source: BMC Med. 2022 Jan 26;20:21. doi: 10.1186/s12916-021-02219-7 (PMC8790875; doi:10.1186/s12916-021-02219-7)
Supplement: Supplementary file 1 — Additional file 1: Table S1. Clinical characteristics of patients with HFpEF and non-HF patients using ESC criteria in HCM. [file 12916_2021_2219_MOESM1_ESM.docx]

**Table S1. Clinical characteristics of patients with HFpEF and non-HF patients using ESC criteria in HCM.**

| Parameters | All (n=1178) | HFpEF^#^ (n=761) | Non-HF^#^ (n=417) | P-value^*^ |
| --- | --- | --- | --- | --- |
| Age at evaluation (y) | 49$\pm$14 | 50$\pm$14 | 47$\pm$15 | 0.001 |
| female | 415 (35.2) | 302 (39.7) | 113 (27.1) | <0.001 |
| MWT (mm) | 23 (20-26) | 23 (20-27) | 21 (18-26) | <0.001 |
| LVEF(%) | 68$\pm$8 | 69$\pm$6 | 68$\pm$6 | 0.001 |
| LVedd (mm) | 44$\pm$6 | 43$\pm$6 | 44$\pm$5 | 0.004 |
| Unexplained syncope | 149 (12.6) | 108 (14.2) | 41 (9.8) | 0.030 |
| Atrial fibrillation | 230 (19.5) | 175 (23.0) | 55 (13.2) | <0.001 |
| Maximal LVOT gradient | 44 (10-80) | 59 (20-87) | 16 (8-60) | <0.001 |
| NT-proBNP | 988.0 (494.1-1934.4) | 1239.3 (625.6-2277.0) | 626.8 (277.7-1260.9) | <0.001 |
| NYHA class |  |  |  |  |
| I | 385 (32.7) | 0 (0.0) | 385 (92.3) | <0.001 |
| II | 514 (43.6) | 495 (65.0) | 19 (4.6) | <0.001 |
| III/IV | 279 (23.7) | 266 (35.0) | 13 (3.1) | <0.001 |
| Medicine treatment |  |  |  |  |
| Beta-blockers | 861 (73.1) | 568 (74.6) | 293 (70.3) | 0.105 |
| ACEI/ARBs | 232 (19.7) | 124 (16.3) | 108 (25.9) | <0.001 |
| Calcium-channel blocker | 230 (19.5) | 137 (18.0) | 93 (22.3) | 0.075 |
| Diuretic | 269 (22.8) | 205 (26.9) | 64 (15.3) | <0.001 |

Values are presented as the mean±SD, median (interquartile range) or n (%).

^#^ Heart failure phenotypes were identified using ESC criteria.

* Comparison between patients with HFpEF and non-HF patients.

In our cohort, the data on the maximal LVOT gradient were not available in 15 (1.3%) patients and the missing values were imputed using the median.

ESC, European Society of Cardiology; LVedd, left ventricular end-diastolic dimension; LVEF, left ventricular ejection fraction; LVOT, left ventricular outflow tract; MWT, maximal wall thickness; NYHA, New York Heart Association;
