# Supplementary material for: Identification of heart failure with preserved ejection fraction helps risk stratification for hypertrophic cardiomyopathy
Source: BMC Med. 2022 Jan 26;20:21. doi: 10.1186/s12916-021-02219-7 (PMC8790875; doi:10.1186/s12916-021-02219-7)
Supplement: Supplementary file 2 — Additional file 2: Figure S1-S3. FigS1- Kaplan-Meier curve for patients with HFpEF and non-HF patients using ESC criteria in HCM. FigS2- Hazard ratio of HFpEF versus non-HF using ESC criteria in 1178 patients with HCM. Fig S3-Hazard ratio of HFpEF versus non-HF in 613 HCM patients without septal reduction therapy. [file 12916_2021_2219_MOESM2_ESM.docx]

**
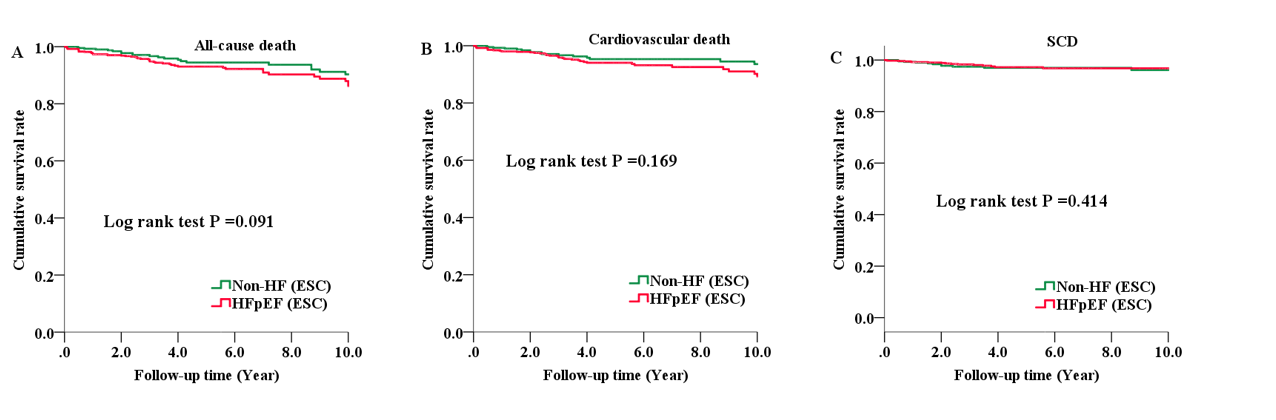
Figure S1.** Kaplan-Meier curve for patients with HFpEF and non-HF patients using ESC criteria in HCM.

Cumulative survival rate curves for all-cause death (A), cardiovascular death (B), and SCD (C).

ESC, European Society of Cardiology; HCM, hypertrophic cardiomyopathy; HFpEF, heart failure with preserved ejection fraction; SCD, sudden cardiac death.

**
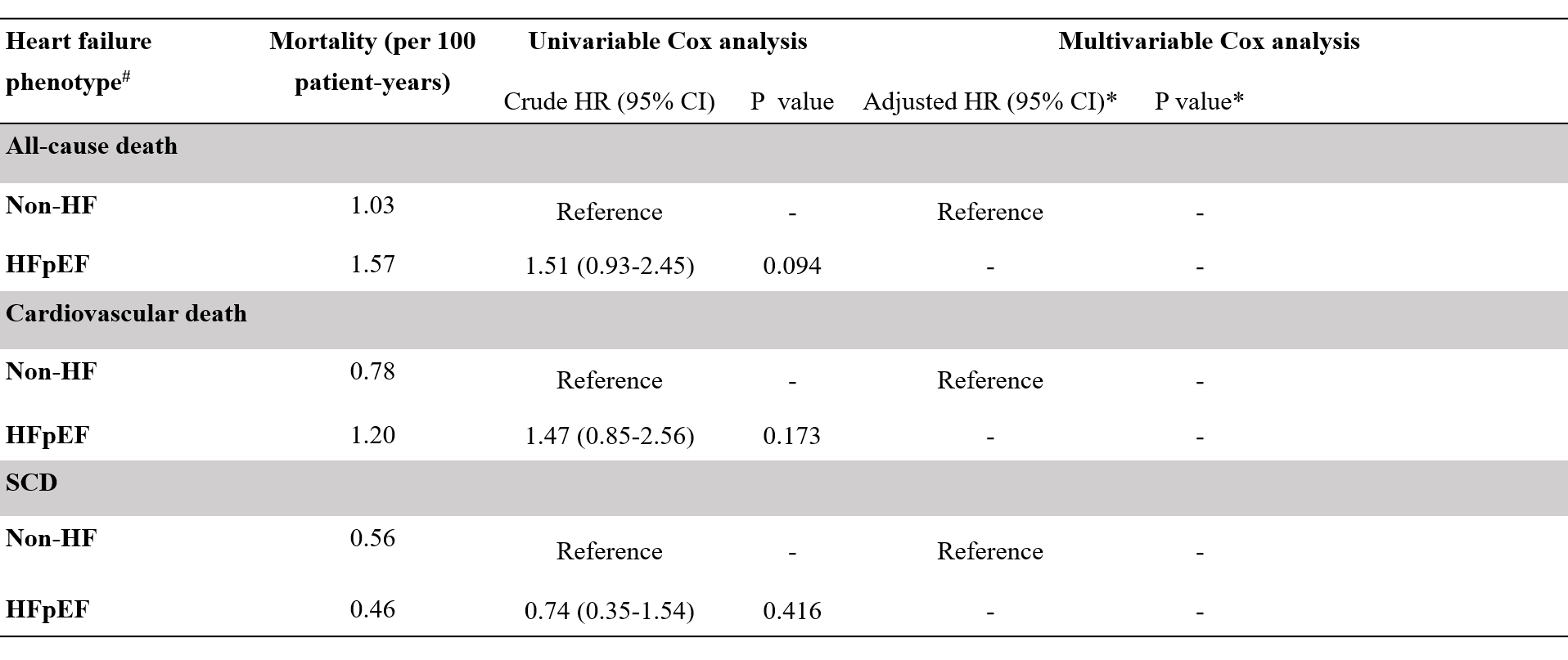
Figure S2.** Hazard ratio of HFpEF versus non-HF using ESC criteria in 1178 patients with HCM.

^#^Heart failure phenotypes were identified using ESC criteria.

*Multivariable Cox regression analysis, models were adjusted for the following covariates using a backward method: age, sex, maximal left ventricular wall thickness, atrial fibrillation, maximal left ventricular outflow tract gradient, and NYHA class.

CI, confidence interval; ESC, European Society of Cardiology; HFpEF, heart failure with preserved ejection fraction; non-HF, patients without heart failure; HR, hazard ratio; SCD, sudden cardiac death.


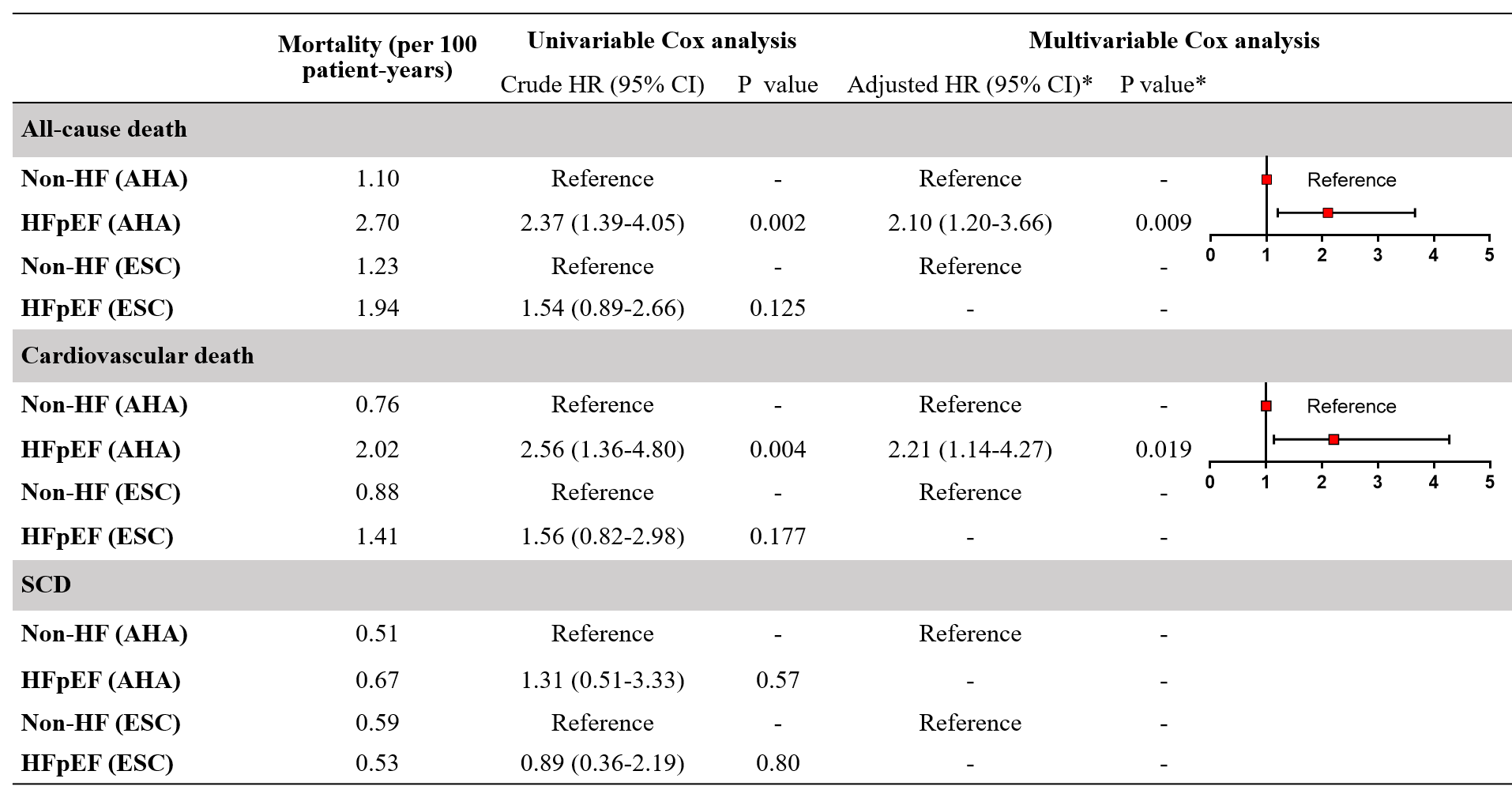
**Figure S3.** Hazard ratio of HFpEF versus non-HF in 613 HCM patients without septal reduction therapy.

AHA, American Heart Association; CI, confidence interval; ESC, European Society of Cardiology; HFpEF, heart failure with preserved ejection fraction; non-HF, patients without heart failure; HR, hazard ratio; SCD, sudden cardiac death

*Multivariable Cox regression analysis. Models were adjusted for the following covariates using a backward method: age, sex, maximal left ventricular wall thickness, atrial fibrillation, maximal left ventricular outflow tract gradient, and NYHA class.
